# Opioid-free anesthesia for postoperative recovery after video-assisted thoracic surgery: A prospective, randomized controlled trial

**DOI:** 10.3389/fsurg.2022.1035972

**Published:** 2023-01-06

**Authors:** Xu-ru Wang, Xiao-yu Jia, Yan-yu Jiang, Zhen-ping Li, Qing-he Zhou

**Affiliations:** ^1^ Anesthesia Medicine, Zhejiang Chinese Medical University, Hangzhou, China; ^2^ Department of Anesthesiology and Pain Medicine, The Affiliated Hospital of Jiaxing University, Jiaxing, China; ^3^ Anesthesia Medicine, Bengbu Medical College, Bengbu, China

**Keywords:** opioid-free, anesthesia, analgesia, postoperative recovery, thoracic surgery

## Abstract

**Purpose:**

Opioid-based anesthesia is a traditional form of anesthesia that has a significant analgesic effect; however, it can cause nausea, vomiting, delirium, and other side effects. Opioid-free anesthesia with dexmedetomidine and lidocaine has attracted widespread attention. This study aimed to compare the effects of opioid-free and opioid-based anesthesia (OFA and OBA, respectively) on postoperative recovery in patients who had undergone video-assisted thoracic surgery.

**Methods:**

Eighty patients undergoing video-assisted thoracic surgery were assigned to receive either opioid-free anesthesia (OFA group) or opioid-based anesthesia (OBA group) according to random grouping. The primary outcome of the study was the quality of recovery-40 scores (QoR-40) 24 h postoperatively. The secondary outcome measure was numerical rating scale (NRS) scores at different times 48 h postoperatively. In addition to these measurements, other related parameters were recorded.

**Results:**

Patients who received opioid-free anesthesia had higher QoR-40 scores (169.1 ± 5.1 vs*.* 166.8 ± 4.4, *p *= 0.034), and the differences were mainly reflected in their comfort and emotional state; however, the difference between the two groups was less than the minimal clinically important difference of 6.3. We also found that the NRS scores were lower in the OFA group than in the OBA group at 0.5 h (both *p < *0.05) and 1 h (both *p < *0.05) postoperatively and the cumulative 0–24 h postoperative dosage of sufentanil in the OBA group was higher than that in the OFA group (*p = *0.030). There were no significant differences in postoperative nausea and vomiting (PONV) (*p = *0.159). No surgical or block complications were observed between the groups.

**Conclusion:**

Opioid-free analgesia potentially increased the postoperative recovery in patients who underwent video-assisted thoracic surgery.

**Trial registration:**

The study protocol was registered in the Chinese Clinical Trial Register under the number ChiCTR2100045344 (http://www.chictr.org.cn/showproj.aspx?proj=125033) on April 13, 2021.

## Introduction

Thoracoscopic surgery is currently the preferred treatment for patients undergoing pulmonary surgery because of its low trauma rate, quick recovery, and high patient satisfaction. As a powerful analgesic, opioids inhibit the sympathetic response and maintain hemodynamic stability during anesthesia, and their anti-injury effect is the core of traditional balanced anesthesia (1). However, side effects and drug abuse caused by opioids have raised widespread concern (2, 3). Perioperative opiates produce analgesic effects, nausea and vomiting, respiratory depression, delirium, hyperalgesia, and other side effects (4, 5), thus prolonging hospital stay and delaying recovery. As a result, opioid-free anesthesia (OFA) using ketamine, lidocaine, dexmedetomidine, and NSAID is gaining attention (6). The enhanced recovery after surgery protocol has also proposed a treatment strategy for multi-mode analgesia to reduce opioid use and its associated side effects (7, 8). As the core of multi-mode analgesia, it advocates for an opiate-thrift technique of using less or no opiates to achieve effective analgesia.

Currently, the simple definition of OFA is as follows: no opioid drugs are used during the operation until the patient wakes up, and opiates are routinely used for postoperative analgesia (9). There are many recent reports on opioid-free anesthesia, including in breast, shoulder, colectomy, laparoscopic cholecystectomy, and gynecological surgery, all of which revealed positive promoting effects on the postoperative recovery of the patients (10–14). Some retrospective studies have also demonstrated the feasibility and effectiveness of OFA in thoracic surgery, reducing the amount of morphine consumption and reducing the postoperative pain scores (15, 16).

The quality of recovery-40 scores (QoR-40), a 40-item questionnaire, is a reliable scale for evaluating the quality of postoperative recovery of patients. The QoR-40 provides a broad and effective assessment of the patient quality of recovery after anesthesia and surgery (17, 18). To date, no prospective studies have used the QoR-40 to evaluate the quality of recovery from non-opioid anesthesia.

Therefore, we hypothesized that OFA could effectively improve postoperative analgesia and reduce the postoperative demand for opioids as well as conventional opioid anesthesia, thus reducing postoperative hyperalgesia and persistent pain. In this study, the primary and secondary outcomes were the QoR-40 score at 24 h postoperatively and the postoperative analgesic effects of the two groups, respectively. Therefore, we compared the effects of opioid-free anesthesia with those of conventional opioid-based anesthesia on postoperative quality of recovery in patients undergoing thoracic surgery.

## Materials and methods

### Study design and participants

This study was conducted in strict compliance with medical ethics and approved by the Ethics Committee of the affiliated hospital of Jiaxing University with the reference number LS2020-297. Informed written consent was obtained from all patients.

We recruited patients aged 18–80 years with ASA physical status I–III who underwent elective thoracoscopic surgery under general anesthesia at our hospital between August and December 2021. All patients were willing to provide informed consent to participate in this trial and were required to complete the QoR-40 test during the preoperative evaluation. The exclusion criteria included a body mass index (BMI) > 30 kg/m^2^, severe hepatic insufficiency (defined as a prothrombin ratio lower than 15), coagulation dysfunction, abnormal heart conduction system (sinoatrial, atrioventricular, or intraventricular block), skin infection from the nerve block, history of taking analgesics and psychotropic drugs for chronic pain, allergy to local anesthetics and general anesthetics, urgent surgery, and refusal to participate.

### Patient grouping, randomization, and blinding method

After entering the operating theatre, patients were randomly divided into two treatment groups, the OFA and OBA groups, at a 1:1 ratio (40 cases in each group). Randomized numbers were generated from https://www.random.org and stored in sealed, opaque envelopes. All patients were blinded to the group allocation. An anesthesiologist was responsible for the patient-administered anesthesia on the day of surgery and collected the intraoperative data. Another anesthesiologist, who was blinded to the grouping, collected the postoperative data in the surgical ward.

### General anesthesia technology

All patients routinely fasted for 6–8 h and abstained from drinking for 2–4 h before surgery. After entering the operating room, electrocardiography, invasive blood pressure, pulse oximetry, and temperature measurements were routinely monitored. Pre-oxygenation was administered for 3 min before anesthesia induction. In the OFA group, a loading bolus of dexmedetomidine 0.5 μg/kg was administered over 10 min, followed by midazolam 0.05 mg/kg, lidocaine 1 mg/kg, propofol 1–2 mg/kg, and rocuronium bromide 0.6 mg/kg. General anesthesia was maintained with propofol [50–150 μg/(kg·min)], sevoflurane (MAC 1.0–1.4), dexmedetomidine [0.2–0.7 μg/(kg·h)], and lidocaine [2 mg/(kg·h)]. In the OBA group, patients received midazolam 0.05 mg/kg, sufentanil 0.5 μg/kg, propofol 1–2 mg/kg, and rocuronium bromide 0.6 mg/kg for induction. General anesthesia was maintained with propofol 50–150 μg/(kg·min), sevoflurane (MAC 1.0–1.4), and remifentanil 0.1–0.25 μg/(kg·min). A double-lumen endotracheal tube was placed 3 min after induction in all patients. Intraoperative muscle relaxation was maintained by intermittent intravenous administration of cisatracurium (3–4 mg per 30 min). The depth of anesthesia was monitored using the bispectral index, which was maintained between 40 and 60 during the operation. Intraoperative mechanical ventilation in volume control mode was performed, and the ventilation parameters were set as follows: tidal volume, 7–8 ml/kg; respiratory rate, 10–12 breaths/min; 70% oxygen concentration; and oxygen flow, 2 L/min. Single-lung ventilation was performed after the incision, and the ventilation parameters were set as follows: tidal volume, 5–6 ml/kg; respiratory rate, 15–20 breaths/min; 100% oxygen concentration; oxygen flow, 2 L/min; PetCO_2_, 35–45 mmHg; and airway pressure, <30 cm H_2_O. After position fixation, all patients underwent an ultrasound-guided thoracic paravertebral nerve block (TPVB) with 20 ml 0.375% ropivacaine. A single dose of NSAIDs (ketorolac 30 mg or parecoxib 40 mg) was administered to all patients before the skin incision. Ephedrine 6 mg or 250 ml of crystalloid solution was administered according to the baseline criteria to maintain the mean arterial blood pressure within 20% of the baseline measurements. All patients were administered granisetron 6 mg intravenously 30 min before the end of surgery to prevent postoperative nausea and vomiting (PONV). All intraoperative maintenance medications were discontinued at the end of the surgery.

### Ultrasound-guided TPVB

Patients were placed in the lateral decubitus position after general anesthesia. A linear array ultrasonic probe was placed vertically along the midline of the trunk. First, the spinous process was identified, and the ultrasonic probe was moved to the operative side until the transverse process, pleura, and rib clearance were visualized. The needle was inserted into the plane along the direction of the ultrasound probe, and the ultrasound image revealed the shadow of the needle. The needle tip entered the paravertebral space to reach the root of the transverse process before the local analgesic (0.375% ropivacaine) was injected into the space. Subpleural pressure was observed during drug injections and the local anesthesia spread in the paraspinal space. We, therefore, chose multi-point or single-point injections according to the position of the incision.

### Surgical procedures

A single or three ports approach was used based on surgical needs and surgeon preference. All patients were placed in the lateral position. For patients with the three ports approach, a 1.5-cm incision after conventional disinfection was made between the seventh costal space in the midaxillary line of the operation side as the operation hole. A 3.0-cm incision was made in the third intercostal axillary line, the skin and subcutaneous tissue were cut, and a protective sleeve was inserted. A 2.5-cm incision was made in the ninth intercostal axillary line as the auxiliary surgical hole. For patients with a single port approach, a 3-cm incision after routine disinfection was made at the 5th intercostal proximal axillary line to cut the skin, subcutaneous tissue, and intercostal muscle. This single hole was used as the operation and observation hole.

### Analgesic protocol and pain evaluation

After surgery, the patient received patient-controlled intravenous analgesia (PCIA): 100 μg sufentanil for a total of 100 ml, no background infusion dose, a self-administered bolus dose of 2 ml, and a locking time of 15 min; then the patient was sent to the post-anesthesia care unit (PACU) for resuscitation. The PCIA lasted for 48 h after the operation. After the patient was awake and the tracheal tube was removed, an anesthesiologist, who was blinded to the grouping, evaluated the pain using a numerical rating scale (NRS) at 0.5, 1, 3, 6, 12, and 24 h postoperatively (0–10 represented different lengths of pain, with 0 indicating no pain and 10 representing severe pain). Preoperative analgesia education was provided to all patients, including using analgesic equipment. The patient needed to press an analgesic pump for rescue analgesia. All patients were asked to complete the QoR-40 questionnaire at 24 h postoperatively.

The patients with massive intraoperative bleeding, who refused to complete the QoR-40 questionnaire, were sent to the ICU after surgery or required an unexpected reoperation after surgery and were eliminated from the study.

### Outcome measures

The primary outcome of the study was the mean difference in QoR-40 scores 24 h postoperatively between the OFA and OBA groups. The QoR-40 is a global measure of the quality of recovery and includes 40 questions and five dimensions of health: physical comfort, emotional state, physical independence, psychological support, and pain (see Supplementary Questionnaire) (17). The secondary outcome measures were the NRS scores of the patients at different times 48 h postoperatively. In addition to these measures, we recorded the mean arterial pressure (MAP) and heart rate (HR) of each patient at different intraoperative times (T0: baseline value, T1: pre-induction of anesthesia, T2: lowest value after induction of anesthesia, T3: maximum value during endotracheal intubation, T4: value at the beginning of the operation, T5: 30 min after the operation, T6: time at the end of the operation, and T7: 30 min after extubation), the dosage of propofol, time to first postoperative analgesic request, postoperative PCIA dosage, PACU duration, PONV (0 = no symptoms, 1 = mild nausea, 2 = severe nausea and vomiting one time, 3 = vomiting two times or more and requiring intravenous antiemetic metoclopramide 10 mg rescue treatment), and postoperative opioid-related adverse effects such as hypotension, respiratory depression, tremors, and urinary retention.

### Sample size calculation

The sample size of this study was calculated using the PASS 15.0 program (NCSS, LLC., Kaysville, UT, United States). According to a pilot study (*n* = 20 in each group, unpublished data), the QoR-40 scores 24 h postoperatively in the OFA and OBA groups were 169.4 ± 6.0 and 166.0 ± 4.1, respectively. Based on the pilot study, a sample size of 72 patients was needed to provide a power of 0.8 and a significance of 0.05. Considering a 10% dropout probability, we recruited 80 patients for the study.

### Statistical analysis

The continuous data were verified and distributed using the Shapiro–Wilk test. The normally distributed data are represented as mean ± standard deviation, and the non-normally distributed data are expressed as median (interquartile range). The normal distribution data were analyzed using an independent sample *t*-test to compare the differences between groups regarding the outcome parameters. Non-normally distributed data were tested using the Mann–Whitney *U* test to analyze the statistical differences between the groups. A generalized estimation equation was used to analyze the difference in pain NRS and hemodynamics (MAP, HR) over time. The chi-square test was used to compare differences between sexes, ASA I/II/III patients, surgical incision locations (left/right), and the number of trochal ports (1 port vs*.* 3 ports). Analyses were performed using SPSS version 25.0 (IBM Corp., Armonk, NY, United States), and *p *< 0.05 were considered significant for the test results presented.

## Results

**Figure 1:**
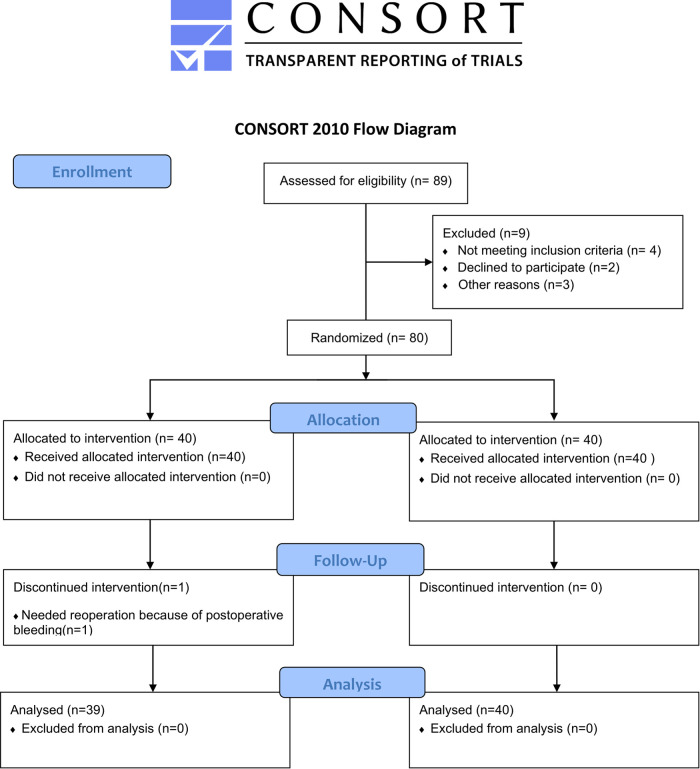
Process chart of this study.

The flowchart of the study is displayed in Figure 1. A total of 89 patients were assessed for eligibility, and 80 patients were included in the study. In the OFA group, one patient required reoperation due to postoperative bleeding. Therefore, 39 patients in the OFA group and 40 in the OBA group were finally enrolled for the analysis. The two groups had similar baseline characteristics (Table 1).

**Table 1:** Descriptive variable characteristics of patients in two groups.

	Group OFA (*n* = 39)	Group OBA (*n* = 40)	*p* value
Age (years)	59 (48–65)	60 (51–65)	0.895
Gender (male/female)	16/23	12/28	0.306
BMI (kg/m^2^)	22.5 ± 3.3	22.9 ± 2.9	0.579
Hypertension	10 (25.6%)	10 (25.0%)	0.948
Diabetes mellitus	3 (7.7%)	4 (10%)	0.718
ASA class I/II/III	6/32/1	7/31/2	0.813
Trochal port (1 port/3 ports)	29/10	25/15	0.257
Surgical incision (left/right)	20/19	14/26	0.144
Type of surgery			0.837
Wedge resection	12 (30.8%)	14 (35.0%)	
Segmentectomy	12 (30.8%)	10 (25.0%)	
Lobectomy	15 (38.4%)	16 (40.0%)	
Pre-QoR-40 score	187.6 ± 2.6	188.5 ± 2.4	0.140

Notes: Data are presented as median (interquartile range), mean ± standard deviation and number of patients (frequency); BMI, body mass index; ASA, american society of anaesthesiologists.

**Figure 2:**
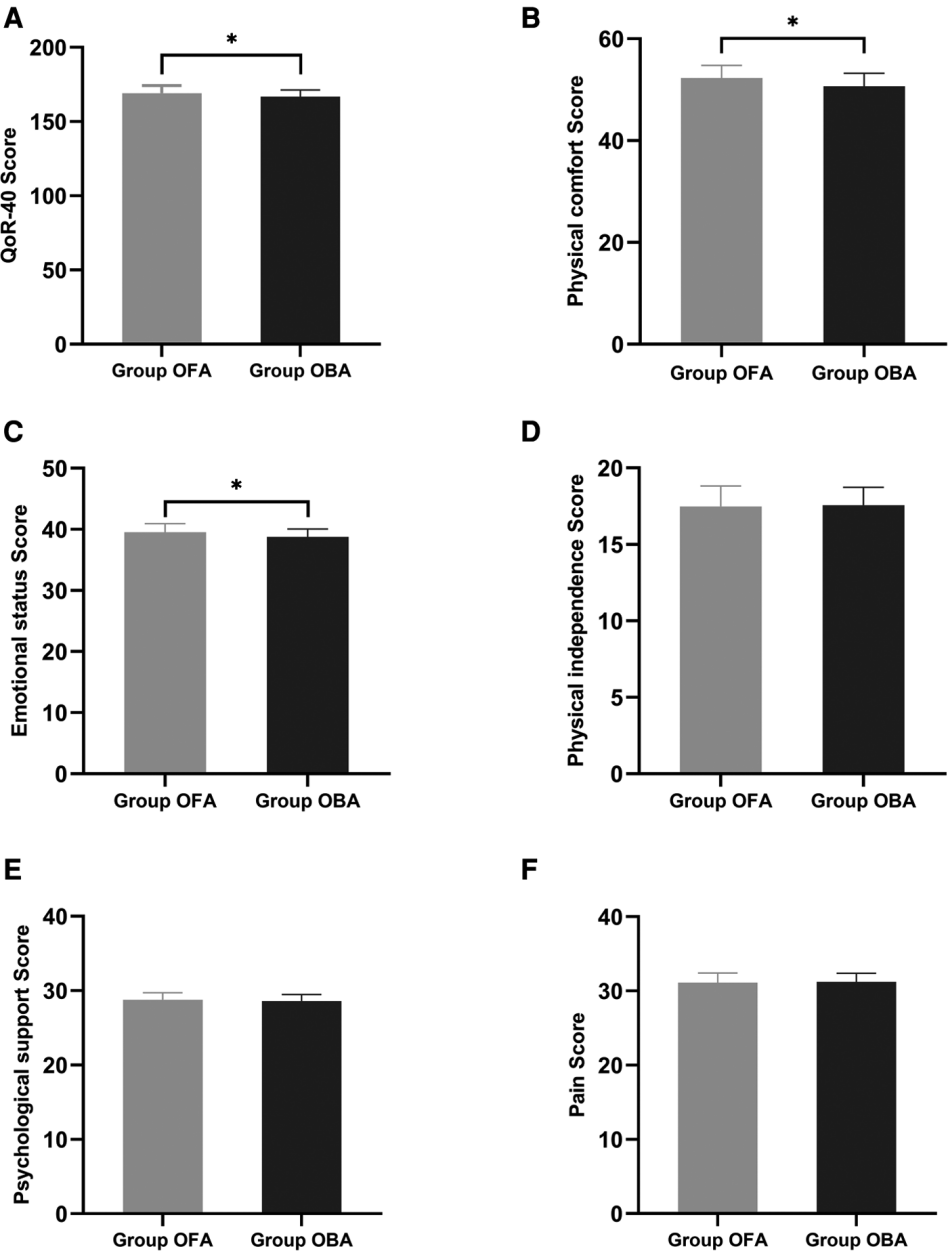
Global and dimension QoR-40 questionnaire score at 24 h post-surgery. Compared to the OBA group, the QoR-40 score was significantly higher in the OFA group (169.1 ± 5.1 vs*.* 166.8 ± 4.4, *p = *0.034). In terms of physical comfort and emotional state, scores in the OFA group are significantly higher than those in the OBA group (physical comfort: 52.3 ± 2.5 vs*.* 50.7 ± 2.5, *p = *0.007; emotional state: 39.5 ± 1.4 vs*.* 38.8 ± 1.3, *p = *0.012), without differences in the other dimensions (both *p > *0.05).

The primary observation index was the QoR-40 scores 24 h after surgery. Compared to the OBA group, the QoR-40 score was significantly higher in the OFA group (169.1 ± 5.1 vs*.* 166.8 ± 4.4, *p = *0.034); however, the difference between the two groups was less than the minimal clinically important difference of 6.3 (19). In terms of physical comfort and emotional state, the scores in the OFA group were significantly higher than those in the OBA group (physical comfort: 52.3 ± 2.5 vs*.* 50.7 ± 2.5, *p = *0.007; emotional state: 39.5 ± 1.4 vs*.* 38.8 ± 1.3, *p = *0.012), while there were no significant differences in the other dimensions (both *p > *0.05) (Figure 2).

The secondary outcome measures were the NRS scores of the patients at 48 h postoperatively. When analyzing the data, we used the generalized estimation equation; the results demonstrated that there was no statistical difference between the two groups in pain NRS scores over time at rest and upon movement at 3, 6, 12, 24, and 48 h postoperatively (all *p > *0.05). However, the pain NRS score was significantly lower in the OFA group than that in the OBA group at 0.5 and 1 h postoperatively while resting or moving (all *p > 0.05*) (Table 2).

**Table 2:** NRS value at different time points of two groups.

NRS value	Group OFA (*n* = 39)	Group OBA (*n* = 40)	*p* value
At rest			
0.5 h	1.0 (1–2)	1.0 (1–2)	0.028^a^
1 h	2.0 (1–2)	2.0 (1–2)	0.015^a^
3 h	2.0 (2–3)	2.0 (2–3)	0.685
6 h	3.0 (2–3)	2.0 (2–3)	0.801
12 h	3.0 (2–3)	3.0 (2–3)	0.593
24 h	2.0 (2–3)	2.5 (2–3)	0.123
48 h	2.0 (2–2)	2.0 (2–2)	0.837
On dynamic			
0.5 h	2.0 (2–2)	2.0 (2–3)	0.026^a^
1 h	2.0 (2–3)	3.0 (2–3)	0.017^a^
3 h	3.0 (3–4)	3.0 (3–4)	0.376
6 h	4.0 (3–4)	3.0 (3–4)	0.829
12 h	4.0 (3–4)	4.0 (3–4)	0.343
24 h	3.0 (3–4)	3.0 (3–4)	0.152
48 h	3.0 (3–3)	3.0 (2–3)	0.678

Notes: Data are presented as median (interquartile range). The differences in NRS value at different intraoperative times were analyzed by generalized estimation equation. NRS at rest in each group at different times showed statistical differences (*p < *0.001); there was no statistical difference in interaction effect (*p = *0.052); NRS on dynamic at different times in each group showed statistical differences (*p < *0.001); there were statistical differences in interaction effect (*p = *0.012).

^a^ Indicates a significant difference between groups.

There was no statistical difference between the OFA and OBA groups in the dosage of propofol and time to the first postoperative analgesic request (*p *= 0.069 and *p = *0.351, respectively). Meanwhile, cumulative sufentanil consumption 24 h postoperatively was significantly lower in the OFA group than in the OBA group (*p = *0.030). The frequency of rescue analgesia requests was significantly lower in the OFA group than in the OBA group (*p = *0.030). There was no statistical difference between the two groups regarding procedure time and PACU duration (*p *= 0.163 and *p = *0.226, respectively) (Table 3).

**Table 3:** Intraoperative anaesthetic dosage, postoperative analgesic, and recovery of two groups.

	Group OFA (*n* = 39)	Group OBA (*n* = 40)	*p* value
Propofol (mg)	400 (300–450)	300 (200–400)	0.069
Procedure duration (min)	80 (60–115)	75 (50–90)	0.163
PACU duration (min)	60 (55–70)	65 (60–79)	0.226
0–24 h postoperative cumulative sufentanil usage (μg)	28 (26–30)	30 (27–32)	0.030^a^
Time to first postoperative analgesic request (h)	2.5 (1–3)	2 (1–3)	0.351
Frequency of rescue analgesic request	4 (2–6)	6 (3–8)	0.030^a^

Notes: Data are presented as median (interquartile range). Abbreviation: PACU, postanesthesia care unit.

^a^ Indicates a significant difference between groups.

**Figure 3:**
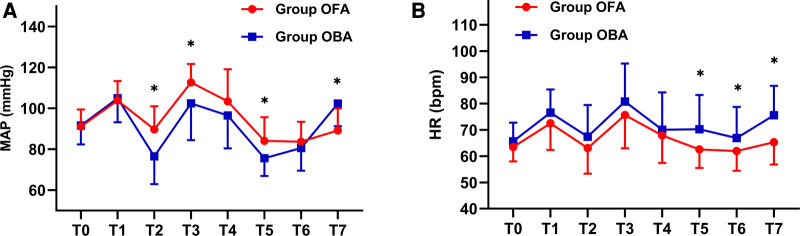
MAP and HR values at different intraoperative times. The differences in MAP and HR at different intraoperative times between the two groups were analyzed with the generalized estimation equation. The MAP in each group at different times and interaction effects are statistically different (both *p < *0.001). The HR in each group at different times and interaction effects are statistically different (*p < *0.001 and *p = *0.012, respectively)*.* T0: baseline value, T1: pre-induction of anesthesia, T2: lowest value after induction of anesthesia, T3: maximum value during endotracheal intubation, T4: value at the beginning of the operation, T5: half an hour after the start of the operation, T6: at the end of the operation, T7: 30 min after extubation. Abbreviation: MAP: mean arterial pressure; HR: heart rate*.* * Indicates a significant difference between groups.

Concerning hemodynamics, the differences in MAP and HR at different intraoperative times were analyzed with a generalized estimation equation, and the results were as follows: the MAP values at T2 (lowest value after the induction of anesthesia), T3 (maximum value during the endotracheal intubation), and T5 (30 m after the beginning of the operation) were significantly higher in the OFA group than those in the OBA group (all *p < *0.05*)*, and the MAP at T7 (30 min after extubation) was significantly lower in the OFA group than that in the OBA group (89.2 ± 10.8 mmHg vs*.* 103.0 ± 11.1 mmHg, *p < *0.05*)*, while there were no statistical differences at the other time points (all *p > *0.05, Figure 3). Meanwhile, HR values at T5 (30 min after the start of the operation), T6 (at the end of the operation), and T7 (30 min after extubation) were significantly lower in the OFA group than those in the OBA group (all *p < *0.05*)*, while there were no statistical differences at other time points (*p > *0.05) (Figure 3). No statistical differences were found in PONV between the two groups (*p = *0.135) (Table 4). No surgical or block complications, including hypotension, respiratory depression, tremors, or urinary retention, were found.

**Table 4:** PONV scores of the two groups.

	Group OFA (*n* = 39)	Group OBA (*n* = 40)	*p* value
PONV scores, *n*			0.159
0	26	20	
1	5	8	
2	4	6	
3	4	6	

Notes: Data are presented as number of patients (frequency); PONV scores (0 = no symptom, 1 = mild nausea, 2 = severe nausea and vomiting 1 time, 3 = vomiting 2 times or more which need rescue antiemetics metoclopramide 10 mg intravenously). Abbreviation: PONV, postoperative nausea and vomiting.

## Discussion

This was the first study to examine the effects of OFA combined with dexmedetomidine and lidocaine on the quality of recovery after thoracoscopic surgery. In this prospective randomized controlled trial, there was a small increase in the QoR-40 scores of patients who received opioid-free anesthesia compared with those of traditional opioid-based anesthesia, which indicated a potential improvement in their quality of recovery.

Our study revealed a statistically significant difference in the QoR-40 scores between the OFA and OBA groups; however, the difference was less than the minimal clinically important difference of 6.3, as previously reported (19). The QoR-40, developed by Myles, is a widely used and extensively validated measure of the quality of recovery of patients (17) and covers all aspects of postoperative recovery. In our study, differences in postoperative recovery quality were mainly reflected in physical comfort and emotional states. We speculated that intraoperative opioid use might increase the risk of PONV. Enhanced recovery after surgery protocols has been successfully achieved in thoracic surgery (7, 20, 21). Some meta-analyses have depicted that intraoperative opioid avoidance effectively reduces PONV (22, 23). Although PONV in the two groups was not statistically significant, the incidence of PONV in the OBA group was higher than that in the OFA group (20/40 vs*.* 13/39). Considering that PONV could affect the comfort and mental state of patients, the QoR-40 score of the OBA group was lower than that of the OFA group in this study, although the difference was minimal.

In terms of postoperative pain, we found that the NRS scores of the OBA group were higher than the OFA group at 0.5 and 1 h postoperatively. Moreover, the consumption of sufentanil and the number of postoperative analgesia requests in the OBA group were higher than those in the OFA group within 24 h after surgery, which was consistent with a retrospective study (16), suggesting a high demand for postoperative analgesia. A review indicated that approximately 0.4% of elderly patients initially treated with opioids continued to receive opioids one year after major surgery, mostly those undergoing thoracic surgery (24). This may be due to the continuous infusion of remifentanil in the OBA group, which might have induced hyperalgesia. Patients receiving large intraoperative doses of remifentanil have a reduced postoperative incision pain threshold (25, 26). Therefore, researchers have considered dexmedetomidine as an operative replacement for remifentanil. Dexmedetomidine not only reduces intraoperative pain sensitivity and the incidence of hyperalgesia but also reduces the postoperative use of opioids (27).

We also recorded the hemodynamic changes at key points during the surgery and demonstrated that patients in the OFA group had a higher MAP during endotracheal intubation and the first half an hour after the operation, while the MAP in the OBA group was significantly higher than that in the OFA group during the recovery from general anesthesia. This may be due to the inability of early opioid-free anesthesia to provide effective analgesia, leading to drastic fluctuations in blood pressure during intense stimulation. However, dexmedetomidine revealed a hypotensive effect during the recovery from anesthesia. In this study, the median HR of the OFA group was lower than that of the OBA group, considering the intraoperative infusion of dexmedetomidine. A clinical study reported that patients treated with dexmedetomidine without opioid anesthesia had more serious adverse events, such as bradycardia and hypoxemia, than patients receiving remifentanil, despite lower postoperative opioid intake (28). However, severe bradycardia was not observed in our study. A randomized controlled study evaluated the change in the pain threshold index and found that OFA with dexmedetomidine was feasible for intraoperative pain management (29). In the present study, intraoperative analgesia levels were not monitored, and the changes in mean arterial pressure during opioid-free anesthesia were not as stable as those under opioid-based anesthesia. Altogether, no serious adverse reactions were observed in this study.

It is worth noting the limitations of this study. First, although we recorded the variation in mean arterial pressure and heart rate at key time points, which helped assess the analgesia at this time, the intraoperative levels could not be accurately established. Second, the total usage of sufentanil was only measured 24 h after surgery, when patients continued to use controlled analgesia for 48 h or longer. Finally, we studied the effect of opioid-free anesthesia on short-term postoperative recovery without tracking its effect on the quality of recovery at 48 h or longer (three months) for chronic pain.

## Conclusions

The results of this study demonstrate the potential beneficial effects of opioid-free anesthesia on postoperative recovery in patients who underwent video-assisted thoracic surgery. Opioid-free anesthesia has attracted increasing attention, and since there is a lack of studies on its effect on the quality of postoperative recovery, this study will provide baseline information for opioid-free anesthesia.

## Data Availability

The raw data supporting the conclusions of this article will be made available by the authors, without undue reservation.
