# Supplementary material for: Opioid-free anesthesia for postoperative recovery after video-assisted thoracic surgery: A prospective, randomized controlled trial
Source: Front Surg. 2023 Jan 6;9:1035972. doi: 10.3389/fsurg.2022.1035972 (PMC9852053; doi:10.3389/fsurg.2022.1035972)
Supplement: Supplementary file 1 [file Datasheet1.docx]

**Supplementary material**

**The Quality of Recovery-40 Questionnaire** **(QoR-40)**

**Part one**

How have you been feeling in the last 24 hours

（This section is based on a scale of 1 to 5. The higher the score, the greater the proportion of the experience in the past 24 hours. 1 = None of the time and 5 = All of the time.）

**Physical comfort** (1=None of the time; 2=Some of the time; 3=Usually; 4=Most of the time; 5=All of the time)

| 1.Able to breathe easily | 1 | 2 | 3 | 4 | 5 |
| --- | --- | --- | --- | --- | --- |
| 2. Have had a good sleep | 1 | 2 | 3 | 4 | 5 |
| 3.Been able to enjoy food | 1 | 2 | 3 | 4 | 5 |
| 4.Feel rested | 1 | 2 | 3 | 4 | 5 |

Total points：

**Emotional status** (1=None of the time; 2=Some of the time; 3=Usually; 4=Most of the time; 5=All of the time)

| 1.Feeling comfortable | 1 | 2 | 3 | 4 | 5 |
| --- | --- | --- | --- | --- | --- |
| 2.Having a feeling of general well-being | 1 | 2 | 3 | 4 | 5 |
| 3.Feeling in control | 1 | 2 | 3 | 4 | 5 |

Total points:

**Psychological support** (1=None of the time; 2=Some of the time; 3=Usually; 4=Most of the time; 5=All of the time)

| 1.Able to communicate with hospital staff (when in hospital) | 1 | 2 | 3 | 4 | 5 |
| --- | --- | --- | --- | --- | --- |
| 2.Able to communicate with family or friends | 1 | 2 | 3 | 4 | 5 |
| 3.Getting support from hospital doctors (when in hospital) | 1 | 2 | 3 | 4 | 5 |
| 4.Getting support from hospital nurses (when in hospital) | 1 | 2 | 3 | 4 | 5 |
| 5.Having support from family or friends | 1 | 2 | 3 | 4 | 5 |
| 6.Able to understand instructions and advice | 1 | 2 | 3 | 4 | 5 |

Total points:

**Physical independence** (1=None of the time; 2=Some of the time; 3=Usually; 4=Most of the time; 5=All of the time)

| 1.Able to go to the lavatory by yourself/ Able to return to work, or usual home activities | 1 | 2 | 3 | 4 | 5 |
| --- | --- | --- | --- | --- | --- |
| 2.Able to write | 1 | 2 | 3 | 4 | 5 |
| 3.Have normal speech | 1 | 2 | 3 | 4 | 5 |
| 4.Able to wash, brush teeth, or shave | 1 | 2 | 3 | 4 | 5 |
| 5.Able to look after your own appearance | 1 | 2 | 3 | 4 | 5 |

Total points:

**Part two**

Have you had any of the following in the last 24 hours?

(the scores for this part ranges from 5 to 1, 5 = None of the time, 1= all of the time)

**Physical comfort** (1= All of the time; 2= Most of the time; 3=Usually; 4= Some of the time; 5= None of the time)

| 1.Nausea | 1 | 2 | 3 | 4 | 5 |
| --- | --- | --- | --- | --- | --- |
| 2.Vomiting | 1 | 2 | 3 | 4 | 5 |
| 3.Dry-retching | 1 | 2 | 3 | 4 | 5 |
| 4.Feeling restless | 1 | 2 | 3 | 4 | 5 |
| 5.Shaking or twitching | 1 | 2 | 3 | 4 | 5 |
| 6.Shivering | 1 | 2 | 3 | 4 | 5 |
| 7.Feeling too cold | 1 | 2 | 3 | 4 | 5 |
| 8.Feeling dizzy | 1 | 2 | 3 | 4 | 5 |

Total points:

**Emotional state** (1= All of the time; 2= Most of the time; 3=Usually; 4= Some of the time; 5= None of the time)

| 1.Had bad dream | 1 | 2 | 3 | 4 | 5 |
| --- | --- | --- | --- | --- | --- |
| 2.Feeling anxious | 1 | 2 | 3 | 4 | 5 |
| 3.Feeling angry | 1 | 2 | 3 | 4 | 5 |
| 4.Feeling depressed | 1 | 2 | 3 | 4 | 5 |
| 5.Feeling alone | 1 | 2 | 3 | 4 | 5 |
| 6.Had difficulty falling asleep | 1 | 2 | 3 | 4 | 5 |

Total points:

**Psychological support** (1= All of the time; 2= Most of the time; 3=Usually; 4= Some of the time; 5= None of the time)

| 1.Feeling confused | 1 | 2 | 3 | 4 | 5 |
| --- | --- | --- | --- | --- | --- |

Total points：

**Pain**（1=All of the time; 2=Most of the time; 3=Usually; 4=Some of the time; 5=None of the time）

| 1.Moderate pain | 1 | 2 | 3 | 4 | 5 |
| --- | --- | --- | --- | --- | --- |
| 2.Severe pain | 1 | 2 | 3 | 4 | 5 |
| 3.Headache | 1 | 2 | 3 | 4 | 5 |
| 4.Muscle pain | 1 | 2 | 3 | 4 | 5 |
| 5.Backache | 1 | 2 | 3 | 4 | 5 |
| 6.Sore throat | 1 | 2 | 3 | 4 | 5 |
| 7.Sore mouth | 1 | 2 | 3 | 4 | 5 |

Total points：
